# Shifts in Respiratory Virus Epidemiology on Reunion Island From 2017 to 2023: Impact of COVID‐19 Pandemic and Non‐Pharmaceutical Interventions

**DOI:** 10.1111/irv.70075

**Published:** 2025-03-05

**Authors:** Nicolas M'nemosyme, Etienne Frumence, Laurent Souply, Diana Heaugwane, Nicolas Traversier, Alizé Mercier, Jamel Daoudi, Jean‐Sébastien Casalegno, Martine Valette, Marie‐Pierre Moiton, Rodolphe Manaquin, Etienne Darieux, Raphaëlle Sarton, Anaïs Grimal, Fabian Thouillot, Xavier Deparis, Bruno Lina, Marie‐Christine Jaffar‐Bandjee

**Affiliations:** ^1^ Laboratoire de Virologie, CHU Félix Guyon Saint‐Denis La Réunion France; ^2^ Centre National de Référence Associé des Virus des Infections respiratoires Saint‐Denis La Réunion France; ^3^ Santé Publique France Réunion Saint‐Denis La Réunion France; ^4^ Laboratoire de Virologie, Institut des Agents Infectieux, Centre National de Référence des virus des infections respiratoires, Hospices Civils de Lyon Lyon France; ^5^ Service de Maladies infectieuses, CHU Félix Guyon Saint‐Denis La Réunion France; ^6^ Service de Maladies infectieuses, CHU GHSR Saint‐Pierre La Réunion France; ^7^ Service de réanimation pédiatrique, CHU Félix Guyon Saint‐Denis La Réunion France; ^8^ Service d'infectiologie pédiatrique, CHU GHSR Saint‐Pierre La Réunion France; ^9^ Agence régionale de santé Réunion Saint‐Denis La Réunion France

**Keywords:** COVID‐19, epidemic, human rhinovirus, influenza, non‐pharmaceutical interventions, respiratory syncytial virus, respiratory viruses, Reunion Island, SARS‐CoV‐2

## Abstract

**Background:**

The COVID‐19 pandemic has reshaped the landscape of respiratory viral infections globally. This study examines these changes on Reunion Island, a French department in the southeastern Indian Ocean.

**Methods:**

Retrospective data from 2017 to 2023, from over 24,000 samples collected across the hospital system, partner laboratories, and a network of sentinel physicians, were analyzed and correlated with the number of consultations at the hospital emergency department and with sentinel physicians for symptoms of acute respiratory infections (ARIs). The epidemiology of respiratory viruses was analyzed by comparing the pre‐ and post‐COVID‐19 periods to assess disruptions in seasonal patterns, changes in virus prevalence, and the affected age groups.

**Results:**

Our database effectively captured the epidemiology of respiratory infections across the island, as demonstrated by its strong correlation with the number of consultations for ARI. Post‐COVID‐19, the influenza virus exhibited multiple epidemic waves within a single year, deviating from its traditional single annual peak and showing a significant decline in circulation from 2020 to 2023. The circulation of respiratory syncytial virus was also impacted post‐COVID‐19, with epidemics starting earlier and lasting longer compared with pre‐COVID‐19 years. Human rhinovirus circulated more prominently in the post‐COVID period, accounting for up to one‐third of positive cases, becoming the most prevalent respiratory virus (excluding SARS‐CoV‐2).

**Conclusions:**

These findings suggest a notable impact of the COVID‐19 pandemic and associated NPIs on respiratory virus circulation on Reunion Island since mid‐2020. They underscore the complex interplay between viral interference, public health interventions, behavioral changes, and youth immunity, emphasizing the need for adaptable strategies in managing respiratory virus outbreaks in the post‐COVID‐19 era.

## 1 Introduction

The emergence of coronavirus disease 2019 (COVID‐19), caused by the severe acute respiratory syndrome coronavirus 2 (SARS‐CoV‐2), in late December 2019 in Wuhan, China, has had a profound impact worldwide [1, 2]. Reunion Island, situated in the southwest of the Indian Ocean and spanning 2500 km^2^ as a French overseas department, is inhabited by around 885,700 people, primarily residing along the coastal areas. On this island, the first case of COVID‐19 was reported on March 11, 2020, at the University Hospital Center (UHC) of La Reunion, in a group of travelers returning from a cruise [3]. Given the lack of available treatments to combat the infection during that period, the government implemented a comprehensive array of public health measures on Reunion Island, similar to those in metropolitan France, in an effort to control the spread of the virus. These measures, referred to as non‐pharmaceutical interventions (NPIs), included a strict lockdown period and the closure of all schools and childcare centers on the island from March 17, 2020, to May 11, 2020. Subsequently, in August 2020, control measures were formally introduced on the island, encompassing social distancing and mandatory mask‐wearing in public. Starting in July 2020, several SARS‐CoV‐2 screening campaigns were launched across all cities on the island, aiming to test all travelers entering and leaving the island, as well as all hospital admissions.

These NPIs were implemented both to reduce the transmission of the virus and to prevent the healthcare system from exceeding its capacity of intensive care beds [4]. Thanks to these measures, Reunion Island registered only 9037 cases and 42 deaths during the initial year of the COVID‐19 pandemic, managing to remain relatively unscathed [5]. The vaccination campaign began on January 15, 2021, and in the subsequent months, a range of new NPIs, including lockdowns and curfews, were introduced. Nevertheless, the island encountered numerous successive waves of infection caused by different variants of SARS‐CoV‐2 (such as Beta, Delta, and Omicron variants) [6]. This led to over 82,796 reported cases in 2021 and a surge to more than 420,850 cases in 2022, according to regional public health authority statistics [6, 7]. The year 2023, on the other hand, was a year of low SARS‐CoV‐2 circulation on the island [8].

It has been reported that the combination of SARS‐CoV‐2 circulation and NPIs across the world has had a significant impact on the circulation of other respiratory viruses [9, 10, 11, 12, 13, 14]. Notably, a global decline in influenza cases and a shift in the seasonal transmission pattern of respiratory syncytial virus (RSV) were evident worldwide. In mainland France, no influenza outbreaks were reported for the 2020–2021 season following the implementation of NPIs, while the RSV bronchiolitis outbreak in children was delayed by 3 months [15, 16].

Reunion Island represents an ideal observatory for monitoring the spread of SARS‐CoV‐2, its evolution in relation to NPIs, and its impact on the circulation of other respiratory viruses within a subtropical, closely‐knit environment with the presence of dense urban areas. This French overseas region in the Southern Hemisphere benefits from a robust surveillance network in a geographic area with a critical lack of data. On this territory, the COVID‐19 pandemic seems to have also impacted the circulation of respiratory viruses, as the epidemiology of severe community‐acquired pneumonia has changed, with a significant decrease in the number of cases in 2020–2021 and no cases of pneumonia related to influenza viruses [17]. To gain a better understanding of how the COVID‐19 pandemic has affected the circulation of influenza virus, RSV, and other respiratory viruses on Reunion Island, we compared the prevalence of these viruses before (2017–2019) and after the pandemic (2020–2023). This study relied on retrospective epidemiological data collected at Reunion UHC from tests conducted within the hospital environment and through the sentinel physician network, which monitors respiratory virus activity across the entire island.

## 2 Methods

### 2.1 Data Collection

The data presented herein were obtained from the virology laboratory of the UHC of La Reunion, covering the period from January 2017 to December 2023. This hospital group receives the majority of respiratory screening samples from the island. Detection tests for respiratory viruses were performed on samples collected from patients presenting with acute respiratory infection (ARI), defined as a sudden onset of fever (≥ 38°C) and cough, which may be accompanied by other symptoms such as breathing difficulties or headache, at the emergency departments of the two main hospital sites located in northern and southern Reunion Island, as well as from hospitalized patients and those tested during outpatient consultations at the hospital's day clinic. As the reference virology laboratory on the island, the UHC laboratory also receives samples from several private laboratories for additional testing.

Additionally, monitoring of seasonal respiratory infectious diseases is conducted through a network of 40 volunteer sentinel general practitioners located throughout the island, coordinated by the French Public Health Agency, Santé Publique France (SPF), as previously presented by Brottet et al. [18]. Each week, all participants in the sentinel network are encouraged to collect a nasal swab from the first two patients presenting with ARI symptoms within 3 days of onset, which are then analyzed at the virology laboratory of the UHC of La Reunion.

Hospital emergency departments and volunteer sentinel general practitioners report weekly to the health authorities the total number of consultations for ARI symptoms.

SARS‐CoV‐2 detection tests began in March 2020 following the identification of the first case on the island. By July 2020, regional testing campaigns were initiated. A drive‐through sampling center was established at the UHC, and SARS‐CoV‐2 detection tests were conducted for individuals presenting with suggestive symptoms, as well as for those needing clearance to travel off the island. Additionally, all patients admitted to the UHC were systematically tested. These screening campaigns were specifically targeted at COVID‐19, and no testing for other respiratory viruses was conducted.

It is important to note that a portion of the SARS‐CoV‐2 tests was conducted in private medical laboratories on the island and was not included in this study.

### 2.2 Virus Detection

For SARS‐CoV‐2 detection, the Seegene Allplex SARS‐CoV‐2 assay kit (IVD) was used with the Microlab Nimbus or Starlet instruments from Seegene (Seegene Inc.), followed by an RT‐PCR with a Biorad CFX96 instrument (Bio‐Rad Laboratories, Inc.). Alternatively, an in‐house RT‐PCR method was used on a Roche LC480 instrument (F. Hoffmann‐La Roche Ltd), based on a technique previously described by the National Reference Center for SARS‐CoV‐2 [19].

For the other respiratory viruses, the Allplex Respiratory multiplex panels were used. Panel 1 for influenza virus type A (H1N1pdm09, H3N2) and type B (Victoria and Yamagata lineages), RSV A and B; Panel 2 for adenovirus (ADV), human metapneumovirus (MPV), human enterovirus (HEV), and parainfluenza virus 1, 2, 3, 4 (PIV1‐4); and Panel 3 for human bocavirus (HBOV), human rhinovirus (HRV), and human coronaviruses (CoV NL63, CoV 229E, and CoV OC43) were used. As described above, the viral RNA was extracted using the Microlab Nimbus or Starlet instruments, and the PCR was performed on a CFX96 thermocycler.

For urgent requests involving the detection of influenza virus or RSV, the Xpert Flu/RSV kit on the GeneExpert instrument (Cepheid, USA) was used. This rapid RT‐PCR test delivers results within 1 h and can detect influenza virus A or B and RSV, but it cannot distinguish between their subtypes.

### 2.3 Statistical Analysis and Graphic Representation

Categorical variables were compared using chi‐squared tests. To establish a baseline for comparisons, data from 2017 to early 2020 were grouped as a pre‐COVID reference period. Each virus's proportion of positive cases was then compared year by year (from 2020 to 2023) against this reference period, resulting in multiple chi‐squared tests across years and viruses.

Given the number of comparisons (k = 36), a Bonferroni correction was applied to adjust for potential alpha inflation due to multiple testing, with a corrected significance threshold set at *p* < 0.001.

After correction, significant *p*‐values indicate robust differences between pre‐ and post‐COVID positivity rates for each virus, enhancing the reliability of findings by mitigating false‐positive risks. All statistical analyses and graphics were carried out in R (Version 4.4.2) using the “ggplot2” library and Python (Version 3.12.2) with the scipy and stats modules.

Statistical analysis by age group followed the same approach, with k set to 4 for each group. A Bonferroni correction was applied, adjusting the significance threshold to *p* < 0.01.

Additionally, a Spearman statistical test was conducted to evaluate the correlation between the number of consultations with the number of positive cases in the 2017–2019 period and the 2020–2023 period with a significance threshold of *p* < 0.01.

## 3 Results

### 3.1 Correlation Between Sample Origin and the Number of Consultations for ARI Symptoms

In this study, epidemiological data on respiratory viruses collected at the virology laboratory of the UHC of La Reunion, covering the period from January 2017 to December 2023, were analyzed. During this period, the laboratory tested a total of 24,835 samples for the detection of major respiratory viruses: influenza virus type A and type B, RSV A and B, ADV, human MPV, HEV, parainfluenza virus (PIV) types 1, 2, 3, and 4, HBOV, HRV, and human coronaviruses NL63, 229E, and OC43 (Table 1).

**TABLE 1:** Positivity rates for RSV, influenza virus, parainfluenza virus, coronaviruses (0C43, 229E, NL63), MPV, HRV, HEV, HBOV, and ADV from 2017 to 2023 on Reunion Island, calculated as the proportion of positive cases out of the total number of positive cases for the year. The degrees of significance compared with the pre‐COVID period are indicated with asterisks (*p‐value < 0.001). 2020a corresponds to the period before Week 13 of 2020, and 2020b to the period after Week 13.

		2017	2018	2019	2020a	2020b	2021	2022	2023
Proportion of positive cases (%)	Influenza virus	62.3	58.6	42.2	11.4	0.2*	7.9*	17.7*	26.5*
	RSV	7.2	10.6	11	43.6	8.8*	26.7*	15.3	14.5
	HRV	17.7	13.9	23.6	23.2	74.2*	32.7*	28.3*	29.7*
	ADV	1.5	2.3	5	1.7	5.3	4.4	6.8*	2.8
	Corona	3	5.5	3.3	4.6	6.1	2.5	4.8	6.2*
	HBOV	1.2	2.5	2.2	2.9	2.9	6.9*	4.3*	1.6
	HEV	1.5	1.3	1.8	2.9	1	6.5*	2.8	4.8*
	MPV	1.2	1.1	5	8.7	1.3	4.7	9.8*	6.2*
	Parainfluenza virus	4.3	4.2	5.8	1	0.2*	7.9*	10.1*	7.7*
Total	Overall positivity rate (%)	63.8	41.4	48.7	54.5	28.1	56.4	60.9	45.7
	Total number of positive cases	1295	526	1151	482	524	1579	3528	3579
	Total number of tests	2030	1272	2364	884	1866	2801	5795	7823

**FIGURE 1:**
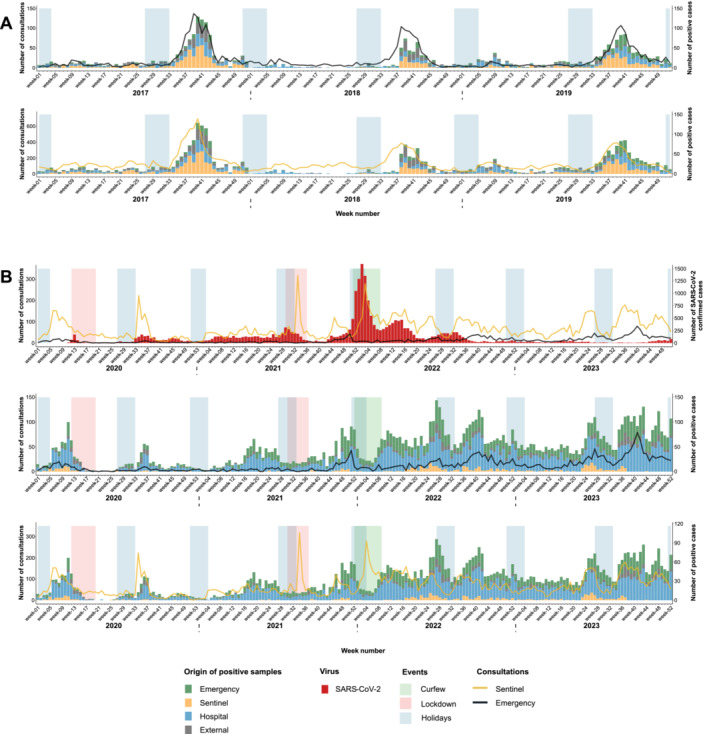
Weekly number of samples from different origins (emergency, sentinel, hospital, and external) on Reunion Island, shown for the period before the COVID‐19 pandemic (January 2017–December 2019) in Panel A, and during the COVID‐19 pandemic (January 2020–December 2023) in Panel B.

These samples were collected either in the hospital emergency department (29% of samples, referred to as *Emergency* in Figure 1) or from other patients tested at the hospital (54% of samples, labeled as *Hospital* in Figure 1), including hospitalized patients and those tested during consultations at the hospital's day clinic. The UHC laboratory also receives samples from several private laboratories for supplementary analysis (11% of samples, identified as *External* in Figure 1). Additionally, patient samples were also obtained from a network of volunteer sentinel general practitioners (6% of samples, referred to as *Sentinel* in Figure 1).

These data were contextualized by comparing them with the number of consultations for acute respiratory symptoms reported in the hospital emergency department and by the network of sentinel general practitioners to assess their representativeness in relation to the epidemiology of respiratory infections on the island.

#### 3.1.1 Pre‐COVID‐19: 2017–2019

From 2017 to 2019, the average number of tests conducted at the virology laboratory of the UHC of La Reunion was approximately 1900 per year, with a positivity rate of around 50% (Table 1). During this period, the number of weekly consultations for ARI closely followed the patterns of respiratory viruses detected, with both emergency and sentinel consultations showing peaks aligned with the typical seasonal waves of these infections (Figure 1A). Consultations from both sources demonstrated a significant correlation with respiratory virus cases detected at the UHC of La Reunion (*p*‐value < 0.01).

#### 3.1.2 COVID and Post‐COVID‐19 Periods: 2020–2023

From 2020 to early 2022, the number of samples collected by the sentinel network decreased (Figure 1B). Due to the COVID‐19 epidemic, sentinel physicians reduced their sample collection and redirected patients to regional testing centers for SARS‐CoV‐2 screening. Sampling gradually resumed afterward. As a result, the proportion of hospital‐based samples also significantly increased post‐COVID‐19.

Consultations for ARI symptoms continued to reflect the varying epidemic waves of respiratory viruses detected at the UHC of La Reunion from 2020 to mid‐2021 (Figure 1B). However, a decline in consultations at emergency departments was observed.

From Week 30 to Week 36 of 2021, when the COVID‐19 incidence rate exceeded 400 per 100,000 inhabitants, a peak in consultations was observed among sentinel physicians, but not at the emergency departments (Figure 1B). This discrepancy can be attributed to lockdown measures that restricted residents to a 10 km radius from their homes, leading them to visit their primary care physicians and the nearest local laboratories rather than the hospital.

At the end of 2021 and the beginning of 2022, during another epidemic wave of SARS‐CoV‐2, the number of consultations for ARI symptoms with sentinel physicians increased, while emergency department consultations remained unchanged despite the high number of SARS‐CoV‐2 positive cases detected at the hospital.

Overall, during the post‐COVID‐19 period (2020–2023), cases of respiratory viruses detected at the UHC of La Reunion significantly correlated with consultations by sentinel physicians and emergency departments for ARIs (*p*‐value < 0.01). Thus, the samples collected during this study appear to provide an accurate representation of the respiratory epidemic situation across all of Reunion Island.

During the period analyzed, from 2017 to 2023, the number of respiratory virus detection tests (excluding SARS‐CoV‐2) significantly increased (Table 1). The laboratory's testing capacity was substantially improved to monitor the progression of the COVID‐19 epidemic. Starting in 2022, the number of tests conducted by the laboratory increased substantially, rising from an average of approximately 2000 tests per year to 5795 and 7823 tests conducted in 2022 and 2023, respectively. This increase can be attributed to a change in the screening strategy at the UHC of La Reunion. To more closely monitor ARIs after the significant COVID‐19 epidemic waves in 2021 and 2022, the number of tests prescribed by hospital physicians to screen for other respiratory viruses increased.

Although the number of positive cases detected has increased, the overall positivity rates for the post‐COVID years are similar to those of the pre‐COVID years (Table 1).

### 3.2 Influenza Virus Circulation From 2017 to 2023

#### 3.2.1 Pre‐COVID‐19: 2017–2019

**FIGURE 2:**
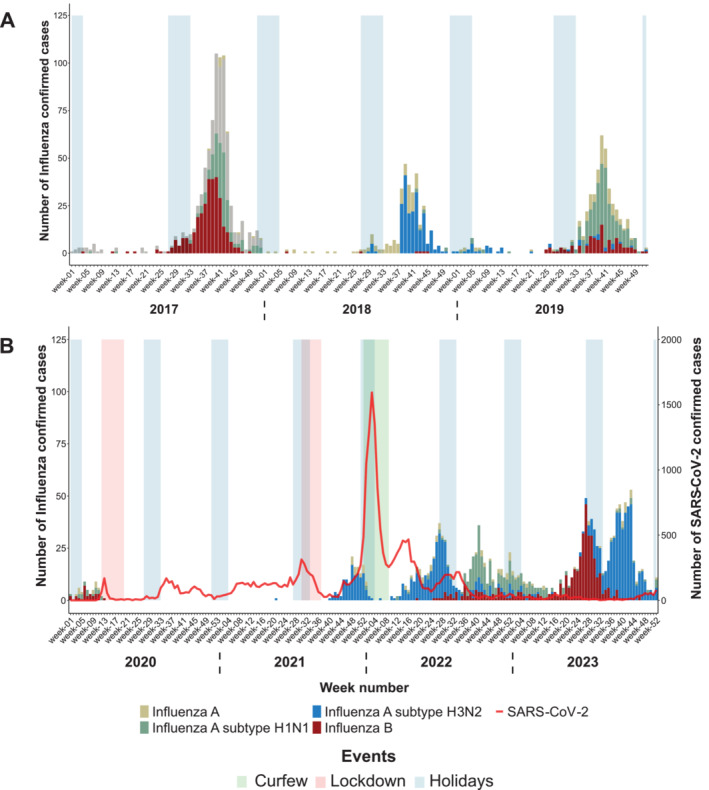
Weekly number of confirmed influenza virus cases on Reunion Island, showing the period before the COVID‐19 pandemic (January 2017–December 2019) in Panel A, and during the COVID‐19 pandemic (January 2020–December 2023) in Panel B. The number of SARS‐CoV‐2 cases recorded at the Reunion UHC was represented by a red line.

From 2017 to 2019, influenza virus circulation on Reunion Island occurred during the austral winter, ranging from July (Week 27) to November (Week 48), with a noticeable epidemic peak observed every year in September–October (Weeks 39–41) (Figure 2A). A secondary peak was also observed in January–February (Weeks 4–7), which was attributed to individuals returning from holidays from mainland France. Each epidemic was characterized by the predominance of a specific influenza virus: type B (Yamagata lineage) in 2017, A(H3N2) in 2018, and A(H1N1)pdm09 in 2019. During this period, influenza viruses were the predominant respiratory viruses on the island, representing 62.3%, 58.6%, and 42.2% of positive cases detected on the island in 2017, 2018, and 2019, respectively (Table 1).

#### 3.2.2 COVID and Post‐COVID‐19 Periods: 2020–2023

The Year 2020 was defined by the emergence of the SARS‐CoV‐2 pandemic. Only 56 confirmed influenza cases were reported by the UHC of La Reunion, all of which were recorded during Weeks 1–13, prior to the emergence of SARS‐CoV‐2 and the implementation of the first lockdown and school closures (Figure 2B). Notably, no influenza virus was detected throughout the rest of the year. In 2021, a resurgence of influenza virus A(H3N2) became evident from week 40 onwards, just 2 weeks after the end of the second lockdown. By the end of 2021, the circulation of the influenza virus was stopped by the combination of the rapid surge in SARS‐CoV‐2 cases, driven by the Delta and Omicron variants, and the implementation of a regional curfew on January 1, 2022. The circulation of the influenza A(H3N2) resumed after the lifting of the curfew at the end of February 2022, despite the occurrence of the second wave of the SARS‐CoV‐2 Omicron variant. Finally, the emergence of A(H1N1) during the 2022 austral winter resulted in an epidemic that peaked in Week 41, in a similar timeframe as observed prior to the COVID‐19 epidemic.

In 2023, two epidemic waves also affected the island, with influenza B at the start and influenza A(H3N2) subsequently, following the same timeframe as 2022. Since the emergence of COVID‐19, the influenza virus is no longer the predominant respiratory virus circulating on the island, and its circulation has significantly decreased (Table 1). The proportion of positive influenza cases observed among positive cases was only 7.9% in 2021, 17.7% in 2022, and 26.5% in 2023.

### 3.3 RSV Circulation From 2017 to 2023

#### 3.3.1 Pre‐COVID‐19: 2017–2019

**FIGURE 3:**
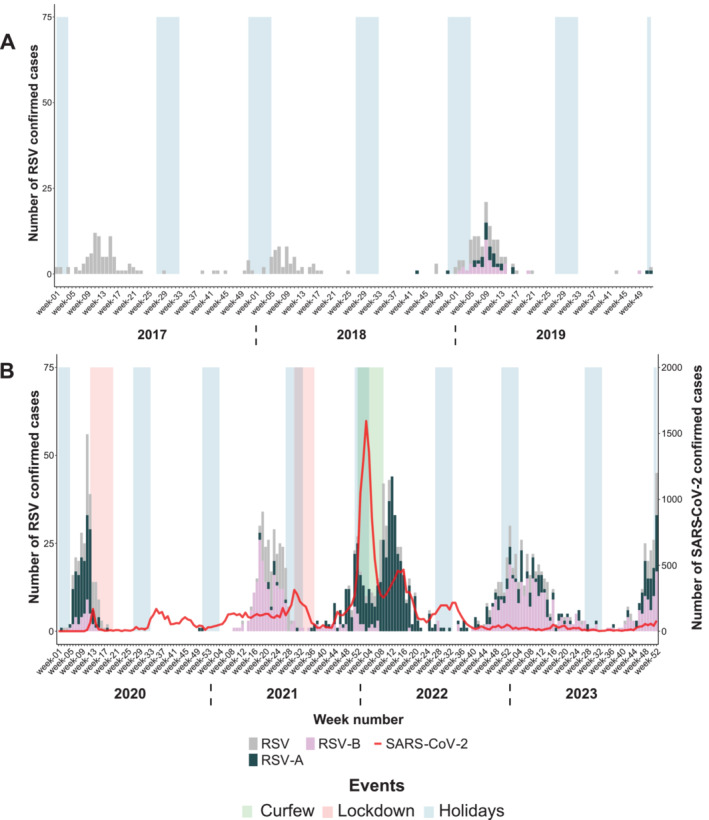
Weekly number of confirmed RSV cases on Reunion Island, showing the period before the COVID‐19 pandemic (January 2017–December 2019) in Panel A, and during the COVID‐19 pandemic (January 2020–December 2023) in Panel B. The number of SARS‐CoV‐2 cases recorded at the Reunion UHC was represented by a red line.

On Reunion Island, there is typically a seasonal circulation of RSV from January to April, with a peak in incidence occurring each year in February–March, coinciding with the austral summer when a hot and humid climate prevails, just after the usual epidemic period observed in mainland France (Figure 3A). From 2017 to 2019, RSV accounted for 7%–11% of the respiratory viruses circulating in Reunion (Table 1).

#### 3.3.2 COVID and Post‐COVID‐19 Periods: 2020–2023

At the beginning of 2020, before the emergence of COVID‐19 on Reunion Island, an RSV epidemic was observed, primarily involving RSV subtype A (Figure 3B). When the first lockdown and school closures were implemented on March 17, 2020, following the initial case of COVID‐19 on Reunion Island, the impact was immediate, leading to a rapid cessation of the circulation of RSV. Apart from two isolated cases, RSV circulation ceased until the end of 2020 (Figure 3B). In 2021, the RSV epidemic, mainly involving RSV B, occurred despite ongoing circulation of SARS‐CoV‐2, with a 12‐week delay as compared with the pre‐COVID period. RSV B circulation continued at a low level throughout the year, with a shift to RSV A observed around Week 48. During 2021, RSV circulated extensively, accounting for 26.7% of positive respiratory virus cases recorded, making it the second most prevalent virus. At the beginning of 2022, a decrease in RSV positive cases was observed during a SARS‐CoV‐2 Omicron epidemic peak. However, a rebound of the RSV cases was observed following the rapid decline in COVID‐19 cases (Figure 3B). Subsequently, RSV continued to circulate at a low level throughout the year 2022, with a resurgence of the epidemic around Week 46 and a shift to RSV B. In 2023, the same temporal kinetics of viral circulation were observed, with RSV B as the predominant variant. The positivity rate of RSV decreased in 2022 and 2023 compared with 2021 and is approaching pre‐COVID values, accounting for 15.3% and 14.5% of positive respiratory virus cases recorded, respectively (Table 1).

### 3.4 COVID‐19 Pandemic Impact on Other Respiratory Viruses

#### 3.4.1 Pre‐COVID‐19: 2017–2019

**FIGURE 4:**
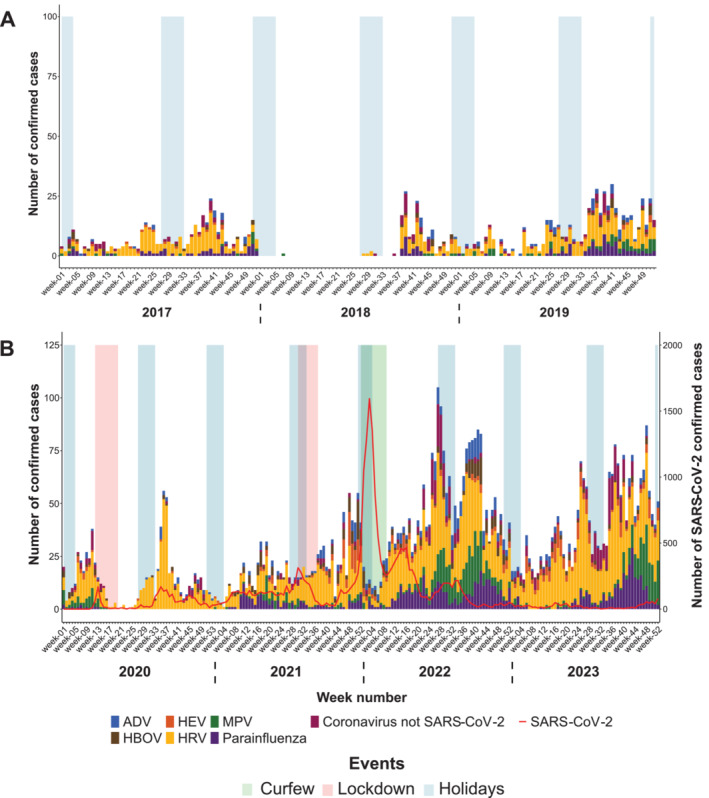
Weekly number of confirmed ADV, HBOV, HRV, MPV, HEV, parainfluenza viruses, and coronaviruses (OC43, NL63, 229E) cases in Reunion Island, showing the period before COVID‐19 (January 2017–December 2019) in Panel A, and during the COVID‐19 pandemic (January 2020–December 2023) in Panel B. The number of SARS‐CoV‐2 cases recorded at the Reunion UHC was represented by a red line.

From 2017 to 2019, on Reunion Island, rhinovirus (HRV) ranked as the second most prevalent virus, circulating throughout nearly the entire year (Table 1 and Figure 4), representing between 13.9% and 23.6% of the positive respiratory virus cases recorded. Other viruses, such as ADV, bocavirus (HBOV), MPV, enterovirus (HEV), and groups of viruses like PIV 1–4, coronaviruses OC43, NL43, and 229E, did not exceed 6% of positive cases during the year. These viruses circulated throughout the year at low levels, with a slight increase observed during the austral winter, between Weeks 35 and 42.

#### 3.4.2 COVID and Post‐COVID‐19 Periods: 2020–2023

In 2020, there was a peak of HRV during Week 35, during the austral winter, occurring in the absence of influenza virus and RSV circulation (Figure 4). That year, after the detection of the first COVID‐19 case, HRV was the predominant respiratory virus detected, representing 74.2% of positive respiratory virus cases recorded (Table 1). Subsequently, in 2021 and continuing into 2022 and 2023, rhinovirus circulated throughout the year, without causing any particular peak during the winter. HRV accounted for 28.3%–32.7% of total positive respiratory virus cases between 2021 and 2023, compared with the usual range of 13.9%–23.6% before the COVID‐19 pandemic (Table 1), making it the most prevalent respiratory virus on Reunion Island. Regarding other respiratory viruses, PIV exhibited a substantial increase starting in 2021, exceeding 7% of all positive cases, compared with the period before the COVID‐19 pandemic (Table 1). These viruses were responsible for two small epidemic peaks in 2022 and a larger peak toward the end of the austral winter in 2023 (Figure 4B). MPV also showed a significant increase, particularly in 2022 and 2023, following the same pattern as PIV (Table 1 and Figure 4B).

### 3.5 Age Groups Affected by Influenza Virus, RSV, and HRV

#### 3.5.1 Influenza Virus

**FIGURE 5:**
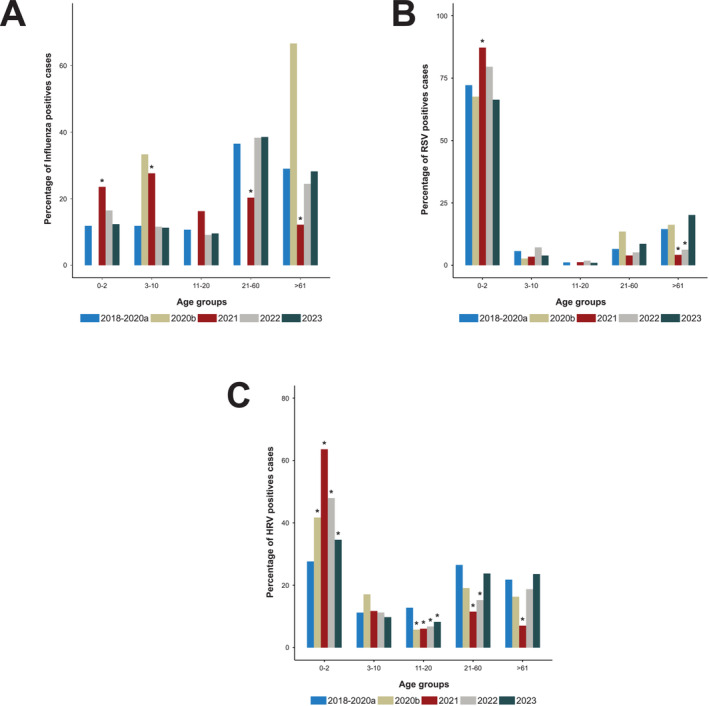
Distribution of positive cases by age group for influenza virus (A), RSV (B), and HRV (C) from 2017 to 2023. The degrees of significance compared with the pre‐COVID period are indicated with asterisks (*p‐value < 0.01).

Between 2017 and 2020, the age groups 21–60 and > 60 were the most affected by influenza virus infection (Figure 5). Starting in 2021, this trend was significantly altered, and influenza predominantly affected individuals under 21 years of age, accounting for 70% of the positive cases (compared with 40% before the COVID‐19 epidemic). In 2022 and continuing into 2023, the age distribution of influenza‐infected cases seems to be reverting to pre‐COVID characteristics.

#### 3.5.2 RSV

Between 2017 and 2020, RSV primarily affected the 0–2 age group, representing up to 70% of cases (Table 1). In 2021, a significant increase in RSV circulation was observed among children under 2 years of age, accounting for 90% of the positive cases. Among the rest of the population, RSV circulated to a lesser extent. In 2022 and 2023, RSV among children under 2 showed a decrease, approaching pre‐COVID values.

#### 3.5.3 HRV

Before the COVID‐19 pandemic, HRV circulated across all age groups of the population (Table 1). Starting from the 16th week of 2020, there was a significant increase in rhinovirus circulation in children under 2 years of age as compared with the pre‐COVID‐19 period. In 2021, children aged 0–2 years were primarily responsible for HRV circulation, accounting for 67% of positive cases compared with 30% before COVID‐19. From 2022 to 2023, the age profile of individuals infected with HRV seems to be reverting to pre‐COVID values.

## 4 Discussion

Since March 2020, COVID‐19 has dominated the landscape of respiratory viral infections worldwide. However, the burden of other respiratory viral diseases has become a matter of crucial concern as COVID‐19 containment measures had been lifted [10, 11, 12, 13, 14]. It is now imperative to closely monitor the changes that have occurred in the prevalence of human viral respiratory diseases locally. In this comprehensive epidemiological study, we have made several observations that shed light on the dynamics of human respiratory viral infections after the emergence of the COVID‐19 pandemic compared with the pre‐COVID‐19 period in Reunion Island. The retrospective data analyzed in this study were derived from samples processed by the virology laboratory of the UHC La Reunion, which may significantly represent the respiratory epidemic situation across the entire island, as compared with the number of consultations for ARI symptoms in the island's emergency departments and among a panel of general practitioners.

Among the major respiratory viruses, influenza viruses and RSV are recognized for exhibiting regular seasonal circulation on a global scale. On Reunion Island, influenza outbreaks typically occur during the austral winter season, spanning from June to November, with peak activity in October. In contrast, RSV epidemics occur during the austral summer season, with a peak observed in March. Epidemics caused by these viruses are typically characterized by the dominance of one subtype at a time.

Since the COVID‐19 pandemic, the seasonality of these two viruses has been completely disrupted. In mainland France, the 2020–2021 winter season saw no influenza epidemic, while the RSV epidemic began 12 weeks later compared with previous seasons [20, 21].

On Reunion Island, there were no epidemics caused by these viruses in 2020 (Figures 2 and 3) as observed in many parts of the world [15, 22, 23]. These results suggest that the effects of the NPIs implemented to stop the circulation of SARS‐CoV‐2 may have also been very effective in stopping the circulation of the other respiratory viruses. In 2021, the circulation of influenza and RSVs resumed on our territory, but with a 10‐ to 12‐week delay in the epidemic. A change in the seasonality of RSV was also observed in Germany, Australia, and South Africa, coinciding with the easing of the NPIs at the end of 2020 [13, 24, 25].

Since 2022, the influenza epidemic on Reunion Island has undergone significant changes, featuring two distinct outbreaks within the same year, each involving different subtypes.

Meanwhile, the 2021–2022 influenza epidemic in France appeared earlier and lasted 25 weeks [20]. Also, the RSV epidemic on Reunion Island began earlier and lasted longer than in the pre‐COVID‐19 years, a phenomenon similarly observed in mainland France from 2021–2022 [26].

On Reunion Island, in late 2021 and early 2022, the emergence of the Omicron variant coincided with a notable decline in cases of both influenza virus and RSV, although the number of tests performed remained stable during this period, suggesting a potential displacement effect where the SARS‐CoV‐2 wave superseded the other epidemics (Figures 2, 3, 4). As soon as the curfew was lifted in 2022, and the number of SARS‐CoV‐2 cases decreased, the epidemics of RSV and influenza virus resumed. While this finding may be incidental, a viral competition mechanism known as viral interference might have occurred between these respiratory viruses and SARS‐CoV‐2 [11, 27]. Evidence of such inhibitory interactions between respiratory viruses was described in France in 2009 during the influenza A(H1N1)pdm09 virus pandemic, where rhinoviruses were shown to delay the circulation of the pandemic influenza virus [28].

In addition to the significant change in the circulation periods, we also observed a notable modification in the proportion of positive cases caused by non‐SARS‐CoV‐2 respiratory viruses. Regarding the influenza virus, even though multiple subtypes have circulated since late 2021, with numerous epidemic peaks, its prevalence and positivity rate have decreased compared with the pre‐COVID‐19 period on Reunion Island. Similar results were observed globally, with influenza virus activity remaining low during the 2021–2022 influenza season worldwide [29]. RSV, on the other hand, has been circulating significantly more in the territory since late 2021. This observation also applies to HRV, which lacks seasonality, as it became the predominant non‐SARS‐CoV‐2 respiratory virus circulating in Reunion Island post‐COVID‐19, according to our results. The latter resumed its circulation immediately following the end of the 2020 lockdown and circulates throughout the year to a significant extent. Around the world, multiple studies have reported comparable results for HRV [11, 28].

One possible explanation is that NPIs might not have impacted the circulation of non‐enveloped viruses, such as HRV, in the same way as they did with enveloped viruses like influenza A and SARS‐CoV‐2. Indeed, multiple studies indicate that the intrinsic characteristics of rhinoviruses and respiratory enteroviruses, including their lack of a viral envelope, resilience on surfaces, and the genomic diversity with multiple co‐circulating strains, may facilitate rapid and sustained infections when NPIs were eased [30, 31]. Another possibility is that NPIs, which include measures like mask usage, improved hand hygiene, social distancing, and restricted travel, might not have significantly impacted infants and children. These groups were indeed the most affected by RSV and HRV locally when schools and daycare centers reopened in mid‐2020. Finally, it is also possible that children and infants who were not immunized during the year 2020 due to lockdowns and NPIs were more susceptible to these infections in 2021–2022, thus contributing significantly to the spread of these viruses. It is indeed hypothesized that children who experience reduced early‐life exposure to infectious agents may become more susceptible to diseases later in life [32, 33]. On a reassuring note, our findings from 2022 to 2023 suggest that on our island, the infection rates among different age groups for the three main viruses—influenza virus, RSV, and HRV—are returning to normal levels, as if the immune debt has been effectively erased.

Caution should be exercised when interpreting these results and assessing the direct role of COVID‐19 and NPIs in the observed changes, as modifications in testing strategies, shifts in public behavior, and environmental or climatic factors may also have influenced the epidemiology of respiratory viruses following the COVID‐19 pandemic on Reunion Island.

Furthermore, we chose to include all samples tested at the virology laboratory of the UHC of Reunion Island in this study, which may represent a limitation of our observational approach. These samples were primarily sourced from symptomatic patients within the hospital setting, with a smaller proportion collected by sentinel physicians or from private laboratories on the island. This sampling strategy could introduce bias into our results, as hospital patients may not be representative of the broader general population. Consequently, certain mildly symptomatic pathogens may be underrepresented in our analysis.

In conclusion, our findings suggest a notable impact of the COVID‐19 pandemic and associated NPIs on the circulation of various respiratory viruses on Reunion Island. The influenza epidemic has occurred in several waves throughout the year, and the RSV epidemic now appears to start earlier and last longer. Additionally, our results underscore the increased circulation of viruses that were less prevalent than influenza in the pre‐COVID period, such as PIVs, MPV, and HEV. This increase has occurred at the expense of influenza, the latter seeing a notable decline in circulation. Moreover, the infection rates among different age groups for the main respiratory viruses are returning to pre‐COVID‐19 levels. These alterations in the epidemiology of these viruses are probably multifactorial, influenced by a combination of factors, including the widespread implementation of several NPIs, reductions in travel, the immune debt, and possible SARS‐CoV‐2 viral interference. Modifications in testing priorities and surveillance systems due to the SARS‐CoV‐2 pandemic may have also influenced the statistics of respiratory viruses.

Describing these changes in our territory throughout the COVID‐19 pandemic offers insights into the complex factors influencing the co‐circulation of respiratory viruses in the community, which can inform the implementation of health measures for future epidemics or pandemics. This will allow health authorities to inform the medical community about these changes and adjust the influenza vaccination strategy and RSV immunization protocols for infants.

## Author Contributions

**Nicolas M’nemosyme:** writing–original draft, data curation, formal analysis, validation, investigation, visualization, software. **Etienne Frumence:** writing–original draft, writing–review and editing, formal analysis, validation, investigation. **Laurent Souply:** investigation, resources. **Diana Heaugwane:** investigation, resources. **Nicolas Traversier:** investigation, resources. **Alizé Mercier:** investigation, resources. **Jamel Daoudi:** investigation, resources. **Jean‐Sébastien Casalegno:** writing–review and editing. **Martine Valette:** writing–review and editing. **Marie‐Pierre Moiton:** investigation, resources. **Rodolphe Manaquin:** investigation, resources. **Etienne Darieux:** investigation, resources. **Raphaëlle Sarton:** investigation, resources. **Anaïs Grimal:** investigation, resources. **Fabian Thouillot:** investigation, resources. **Xavier Deparis:** investigation, resources. **Bruno Lina:** writing–review and editing. **Marie‐Christine Jaffar‐Bandjee:** writing–original draft, conceptualization, methodology, investigation, supervision, funding acquisition, project administration, resources.

## Ethics Statement

Ethical approval was not required for this work as data were obtained within routine surveillance.

## Conflicts of Interest

The authors declare no conflicts of interest.

## Data Availability

The authors have nothing to report.
